# Stabilization of Nanodispersed Cerium Phosphate in Matrices Containing Cellulose Derivatives, Citric Acid and Metronidazole

**DOI:** 10.3390/polym18172079

**Published:** 2026-08-27

**Authors:** Elena L. Chuvilina, Olga I. Andreeva, Akhmedali A. Gasanov, Victor A. Stupin, Vladimir A. Parfenov, Natalia E. Manturova, Ekaterina V. Silina

**Affiliations:** 1 LANHIT LLC., 105118 Moscow, Russia; chuvilina.elena@lanhit.ru (E.L.C.); a.olga@lanhit.ru (O.I.A.); akhmedali@lanhit.ru (A.A.G.); 2 Institute of Biodesign and AI in Medicine, I.M. Sechenov First Moscow State Medical University (Sechenov University), 119991 Moscow, Russia; vladimirparfenov@mail.ru; 3 Department of Surgery, Pirogov Russian National Research Medical University, 117997 Moscow, Russia; stvictor@bk.ru (V.A.S.); manturovanatali@yandex.ru (N.E.M.)

**Keywords:** nanocomposites, methylcellulose, carboxymethylcellulose, cerium phosphate, nanoparticles, cellulose, cerium, citric acid, metronidazole, rare earth elements, hybrid, anisotropic nanoparticles

## Abstract

High-dispersion nanocomposite materials based on cerium(III) orthophosphate stabilized with different forms of cellulose (methylcellulose (MC), carboxymethylcellulose (CMC) and sodium carboxymethylcellulose (CMCNa)) with the addition of citric acid were synthesized by chemical precipitation. Experimental samples modified with metronidazole were obtained. A comprehensive analysis of their phase composition, morphology, and physicochemical characteristics was performed using X-ray diffraction, transmission electron microscopy and IR spectroscopy. According to the results of physicochemical studies all synthesized nanocomposites contained nanocrystals with a bimodal size distribution (width 3–6 nm, length 15–200 nm), depending on the matrix composition, and diffraction peaks characteristic of CePO_4_. IR spectroscopy suggested a mechanism for the formation of chemical bonds: cellulose polymer chains are retained on the CePO_4_ surface mainly through a branched network of weak hydrogen bonds, while the carboxylate groups of citric acid form coordination bonds with Ce^3+^. A positive effect of citric acid on limiting the linear dimensions of CePO_4_ crystallites in the presence of a cellulose matrix was established.

## 1. Introduction

The development of nanocomposite materials based on biocompatible polymer matrices is one of the fastest-growing fields in polymer science, biomedicine and pharmaceuticals [1,2,3]. A promising and actively developing direction is the creation of innovative hybrid materials combining the catalytic and biochemical functionality of rare earth element (REE) compounds with the high specific surface area, lightness and biodegradability of biopolymers [4,5,6,7]. Cerium nanocompounds (in particular, their oxides and phosphates) are of great interest to researchers in the field of tissue regeneration due to their unique antioxidant, bacteriostatic and immunomodulatory properties [5,6,7,8,9,10,11]. The use of uncoated nanoparticles is often limited due to their tendency to aggregate [11,12,13]. The introduction (encapsulation) of nanoparticles into three-dimensional macromolecular polymer matrices allows not only their stabilization due to spatial effects, but also regulation of the release kinetics of therapeutic agents, providing a prolonged effect [1,2,6,12,13]. The construction of organic-inorganic systems, where natural polysaccharides serve as matrices and rare earth element compounds act as functional fillers, is of practical interest to researchers in various fields of science and technology. Lanthanides impart unique catalytic and optical properties to such composites, while the polymer framework provides mechanical strength, biodegradability and delayed release kinetics due to the controlled porosity of the obtained materials [4,5,6,7,13,14].

The basic structural element for the creation of such matrices is cellulose [4,5,6,15,16,17,18,19]. Cellulose is a linear polysaccharide consisting of anhydroglucose units (AGU) linked by β-(1→4)-glycosidic bonds, with a degree of polymerization (DP) of 50–5000 and more. The developed system of intra- and intermolecular hydrogen bonds determines the high crystallinity and insolubility of natural cellulose, which requires its conversion to water-soluble ethers. Chemical modification of AGU hydroxyl groups allows the production of polyelectrolytes, which can be either nonionic (methylcellulose, MC) or anionic (carboxymethylcellulose, CMC) [20,21,22]. Cellulose derivatives are actively used in various fields, including the development of biomedical products and drugs [22,23,24,25,26]. The introduction of inorganic salts into CMC solutions significantly changes the conformation of polymer chains due to the shielding of carboxylate groups. While monovalent cations preserve phase stability, multivalent ions (including lanthanides, in particular, Ce^3+^/Ce^4+^) cause ionic cross-linking of macromolecules. From the point of view of nanomaterial synthesis, coordination of CMC functional groups with metal ions limits their diffusion, localizing nucleation processes and preventing sedimentation and aggregation of the resulting nanocrystals [4,13,27,28,29,30].

Macroporous three-dimensional hydrogels and cryogels based on collagen, alginate and gum arabic are capable of acquiring similarity to the extracellular matrix, retaining moisture and providing gas exchange [31,32,33,34,35,36,37]. Modification of such hydrogel matrices with cerium oxide and phosphate nanoparticles makes it possible to reduce oxidative stress in the wound area due to the pronounced antioxidant activity of the filler [28,37,38,39,40,41,42,43]. Alternatively, bacterial cellulose (BC) can be used, which demonstrates high rehydration capacity and stability of the three-dimensional network [17,23,25,42,44].

During the formation of heterogeneous polymer-inorganic systems, the main chemical processes occur at the phase interface. During the synthesis of CeO_2_/CePO_4_ nanocomposites, the polymer matrix acts as a template, limiting the spatial growth of grains, while charge transfer in the redox cycle Ce^3+^ → Ce^4+^ on the matrix surface provides catalytic oxidase and phosphatase activity of the system [45,46,47,48], blocking pathogen metabolism [8,10,11,28,43,48,49,50]. Similar synthesis principles are used during the mineralization of calcium phosphate phases on a bacterial cellulose matrix (BC-Ce:CaP) [51] and the creation of cement composites for osteogenesis [52,53,54,55]. The incorporation of the rare earth element cerium (Ce) into the lattice of doped hydroxyapatite (GA) changes the parameters of the crystal lattice and the specific surface area of the polymer-mineral framework (Ce-GA), which allows its use for prolonged desorption of doxorubicin [55,56]. At the same time, the macromolecular environment forms a so-called “protein corona” on the nanoparticle surface, determining biological compatibility in the organism’s liquid media [57].

The kinetics of controlled drug release from hydrogels directly depends on the cross-linking density of the polymer network and the degree of its swelling [28]. A comparative analysis of antibiotic diffusion from composite matrices “chitosan–collagen–Ce-GA” showed that the polymer polyelectrolyte network acts as a diffusion barrier, prolonging drug release compared with unmodified mineral carriers. Thus, hydroxyapatite releases 90% of gentamicin within the first 10 min, whereas chitosan–collagen–cerium hydroxyapatite nanocomposites release 80% of gentamicin after two hours [58]. In this regard, the widely known antimicrobial and antiprotozoal drug metronidazole [59,60,61], which is compatible with various excipients, such as mannitol, various forms of cellulose and gums [60,62], can be considered promising.

This work is a continuation of studies on the synthesis methodology of nanosized cerium compounds [38,43,50,63,64,65], modified with cellulose derivatives, citric acid and the antimicrobial agent metronidazole.

The aim of the present study is the chemical co-precipitation of highly dispersed nanocomposite materials based on cerium(III) orthophosphate, stabilized with various forms of cellulose (MC, CMC, CMCNa) with the addition of citric acid, as well as the preparation of experimental samples modified with metronidazole, followed by a comprehensive analysis of their phase composition, morphology and physicochemical characteristics using X-ray diffraction (XRD), transmission electron microscopy (TEM) and IR spectroscopy methods.

The main scientific novelty of the work lies in establishing the regularities of the influence of the nature and concentration of various water-soluble forms of cellulose and tricarboxylic acids on the geometric parameters and aggregative stability of cerium phosphate nanoparticles during their co-precipitation and simultaneous introduction of the drug agent.

## 2. Materials and Methods

### 2.1. Reagents and Materials

The following starting materials and reagents were used in this study: cerium(III) nitrate hexahydrate, Ce(NO_3_)_3_·6H_2_O (99.99%, LANHIT LLC, Moscow, Russia), as the matrix precursor; ammonium dihydrogen phosphate, NH_4_H_2_PO_4_ (“chemically pure for analysis”, 99.5%, Vekton, Moscow, Russia), as the precipitating agent. Water-soluble cellulose derivatives were used as organic modifiers and structure-directing agents: nonionic methylcellulose (MC) with a degree of substitution (DS) of 1.4–2.2, anionic carboxymethyl cellulose (CMC), and its sodium salt (CMCNa) with a degree of substitution of 1.5. Citric acid (Cit) was used to further functionalize the phosphate matrix, while metronidazole was employed as a commercial antibacterial agent. All solutions were prepared using double-distilled water.

### 2.2. Synthesis of CePO_4_ Nanocomposites Modified with Cellulose Derivatives

Highly dispersed composite materials based on cerium(III) orthophosphate were synthesized by the precipitation method at room temperature. This method has been used repeatedly and described in detail in our previous works [10,43,64,65].

To ensure reproducibility of the synthesis conditions, the total concentration of components in the reaction medium was maintained constant, calculated as CeO_2_ (12.5 g/L) and NH_4_H_2_PO_4_ (25 g/L). The concentration of the introduced polymer (MC, CMC, or Na-CMC) was used as the variable parameter and was 2.5, 1.25, 0.625, 0.3125, or 0.156 g/L.

A typical synthesis procedure was as follows. Under continuous stirring (stirring rate of 350 rpm), 100 mL of the initial Ce(NO_3_)_3_ solution (125 g/L calculated as CeO_2_) and the calculated volume of an aqueous solution of the corresponding cellulose derivative (stock solution containing 5 g in 500 mL of water: 250, 125, or 62.5 mL) were successively introduced into the reaction vessel. The total volume was then adjusted to 875 mL with distilled water. Subsequently, 125 mL of NH_4_H_2_PO_4_ solution (100 g/L) was uniformly added above the surface of the resulting mixture by spray dosing over 15 min using a spray nozzle. The pH of the reaction medium during precipitation was approximately 3.0 and was not additionally adjusted.

Immediately after completion of the precipitation process, the resulting cerium (III) phosphate suspension was transferred into graduated cylinders for fractional separation of nanoparticles according to their size in the mother liquor. The upper sol fraction, containing the most stable composite nanoparticles, was separated and used for subsequent physicochemical characterization. To obtain powder samples, the materials were dried. In addition, part of the CMC-based sample series was subjected to thermal treatment (annealing) at 400 °C for 3 h to investigate thermal stability and phase evolution.

**Figure 1:**
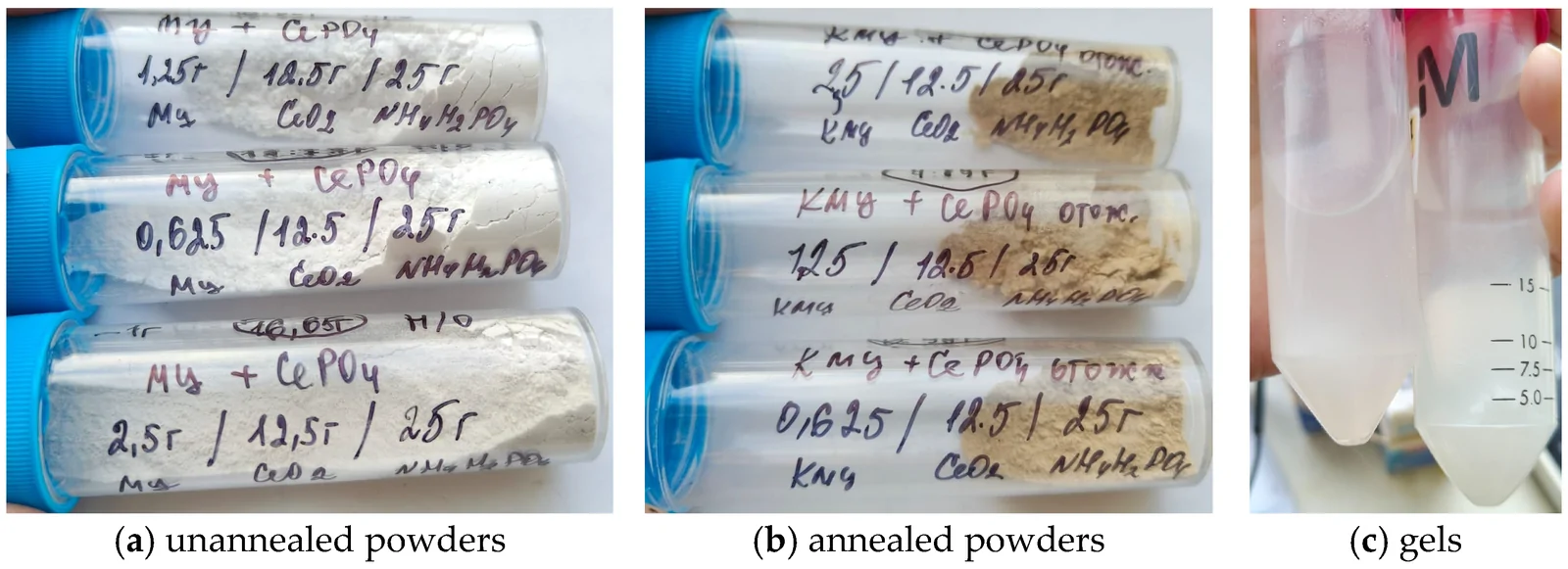
Photographs of nanocomposite samples sent for analysis: (**a**) unannealed powders (methylcellulose used, from top to bottom: 1.25 g, 0.625 g, 2.5 g); (**b**) annealed powders (carboxymethyl cellulose used, from top to bottom: 2.5 g, 1.25 g, 0.625 g); (**c**) gels (from (**a**) on the left, from (**b**) on the right).

The appearance of the unannealed and annealed samples of nanocomposite powders is shown in Figure 1.

### 2.3. Synthesis of Multicomponent CePO_4_ Nanocomposites, Additionally Incorporating Citric Acid and Metronidazole

To obtain multicomponent nanocomposites, the synthesis was carried out in a total reaction volume of 500 mL using a modified procedure with thermal activation. Citric acid (final concentration 12.5 g/L) was introduced into the reaction system containing the cerium precursor and cellulose matrix solutions (final polymer concentration 0.625 g/L) as a modifier of the phosphate matrix, together with a commercial metronidazole preparation in an amount of 500 mg per batch. Precipitation with ammonium dihydrogen phosphate was performed at an elevated temperature (38 °C) under continuous stirring, providing efficient cocrystallization and encapsulation of the drug within the hydrophilic organic–inorganic matrix.

### 2.4. Physicochemical Characterization of the Materials

Transmission electron microscopy (TEM) was performed at the Collective Use Center “Materials Science and Metallurgy” of the National University of Science and Technology MISIS. The morphology, particle size, and degree of nanoparticle agglomeration were investigated using a JEM 2100 high-resolution transmission electron microscope (JEOL, Akishima, Tokyo, Japan) equipped with a LaB_6_ cathode and operated at an accelerating voltage of 200 kV. To ensure high spatial coherence and image resolution, condenser (50 μm) and objective (40 μm) apertures were used. The average electron beam diameter was approximately 100 nm (Spot size 3). Images were acquired after a 2 h thermal stabilization of the instrument. The local crystalline structure was confirmed by electron diffraction (ring diffraction patterns).

X-ray diffraction (XRD). The qualitative and quantitative phase composition, as well as the crystal structure parameters of the obtained nanopowders, were determined by X-ray diffraction using a Bruker D8 diffractometer (Bruker AXS, Karlsruhe, Germany) with monochromatized CuKα radiation (λ = 0.154 nm). Diffraction patterns were recorded in the θ − 2\θ geometry at an operating voltage of 40 kV and a tube current of 40 mA. The crystallite size (coherent scattering domain size) was estimated from the broadening of the diffraction peaks.

Fourier-transform infrared spectroscopy (FTIR, IR) of annealed powders: Intermolecular interactions, the functional composition of the composites, and the incorporation of the therapeutic agent were investigated by Fourier-transform infrared spectroscopy over the wavenumber range of 400–4000 cm^−1^. The measurements were performed using a Bruker VERTEX 70 FTIR spectrometer (Bruker Optik GmbH, Ettlingen, Germany) equipped with a PIKE MIRacle attenuated total reflectance (ATR) accessory (PIKE Technologies, Cottonwood, AZ, USA) with a diamond crystal mounted on a zinc selenide substrate. The spectra were recorded under the following conditions: 32 scans, spectral resolution of 4 cm^−1^, spectral range of 4000–600 cm^−1^, and background spectrum collected immediately before each measurement.

## 3. Results

### 3.1. Phase Composition and Crystal Structure

X-ray diffraction (XRD) analysis of the powders obtained from all the investigated multicomponent systems showed that all samples consisted of single-phase, finely dispersed cerium(III) phosphate. Because the diffraction patterns obtained for the samples were identical, representative XRD patterns for each experimental series are presented in the figures.

**Figure 2:**
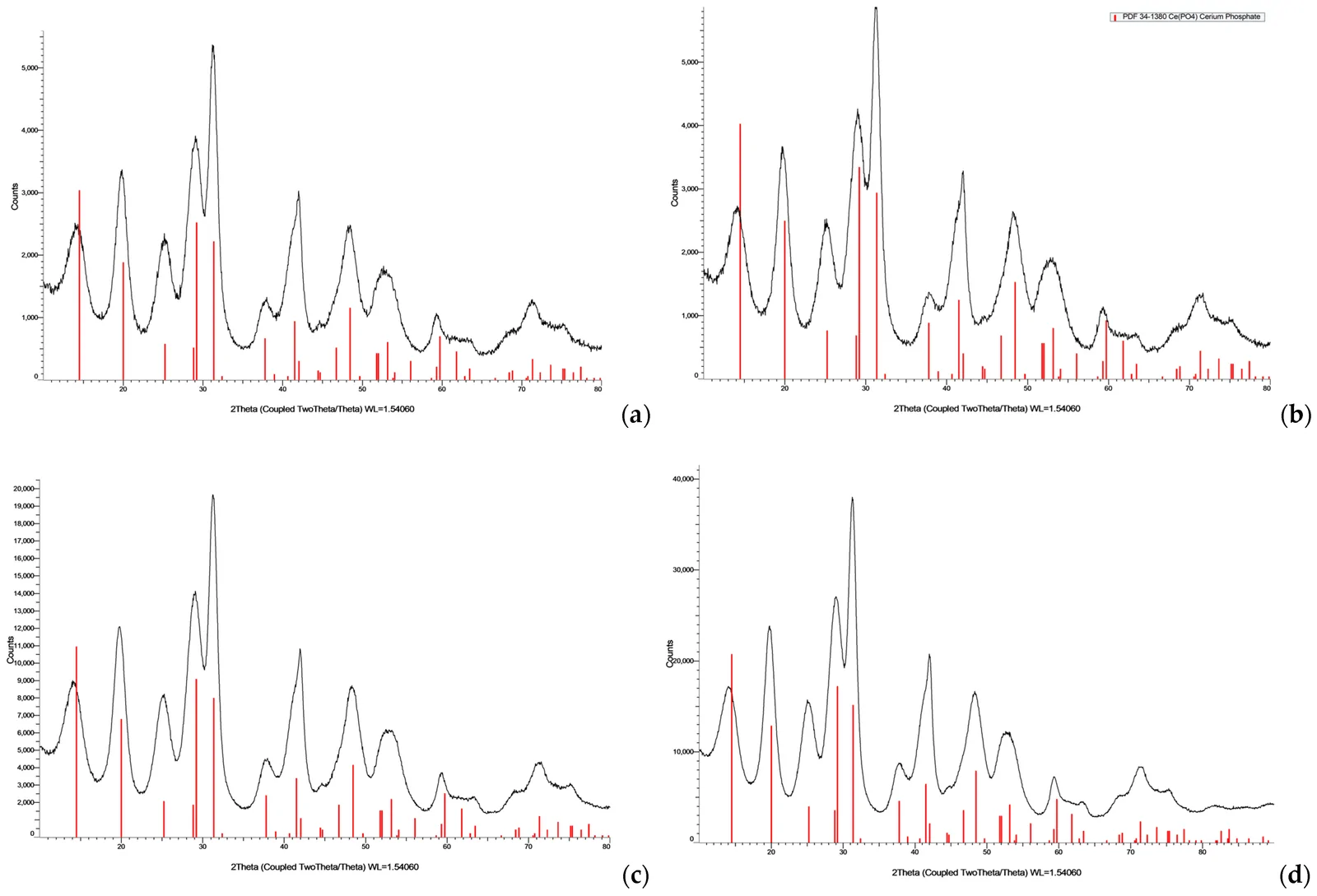
XRD patterns of cerium phosphate nanoparticle samples: (**a**) MC 0.625, (**b**) CMC 0.625, (**c**) CMCNa 0.625, and (**d**) CePO_4_ obtained from a phosphate buffer solution.

**Figure 3:**
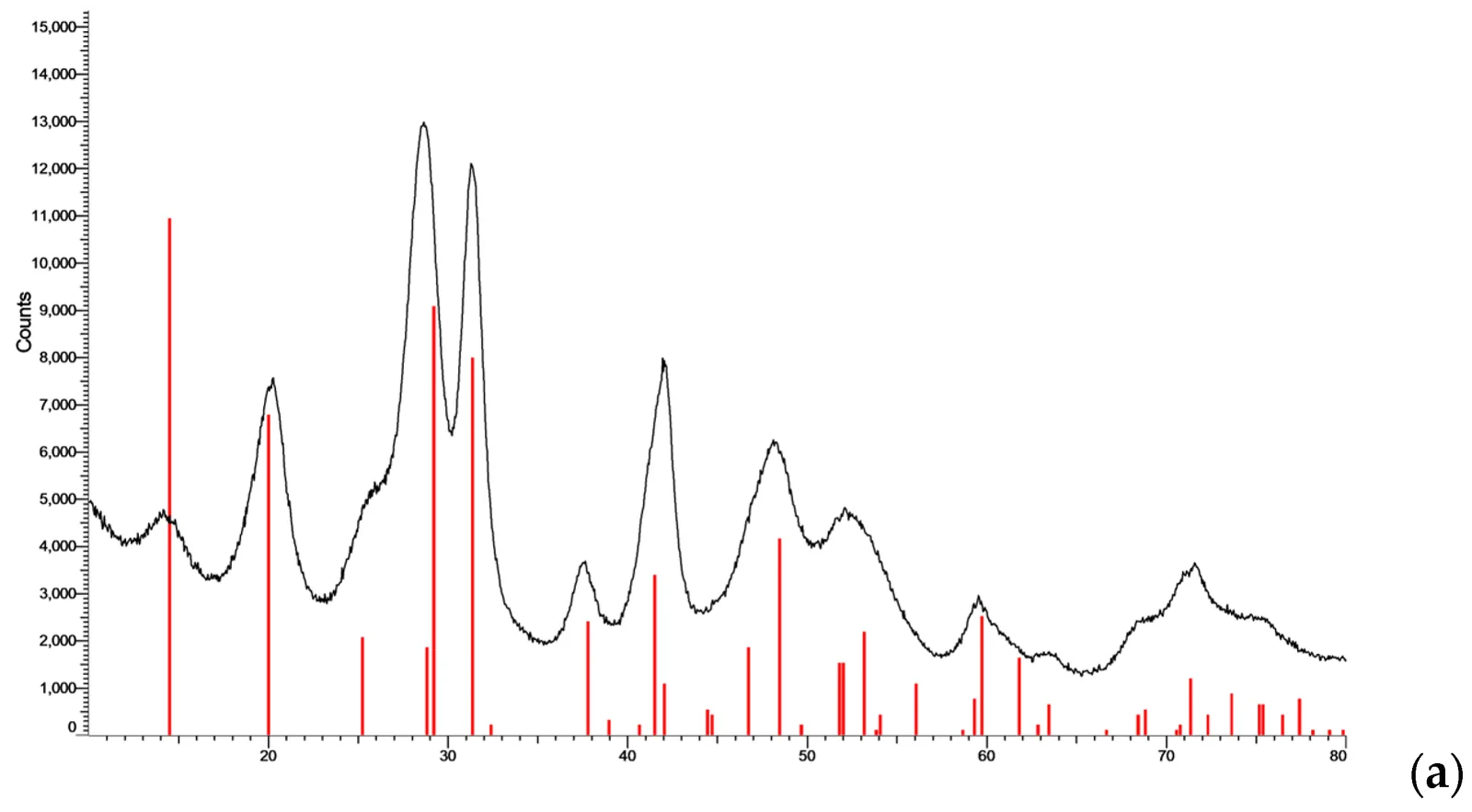

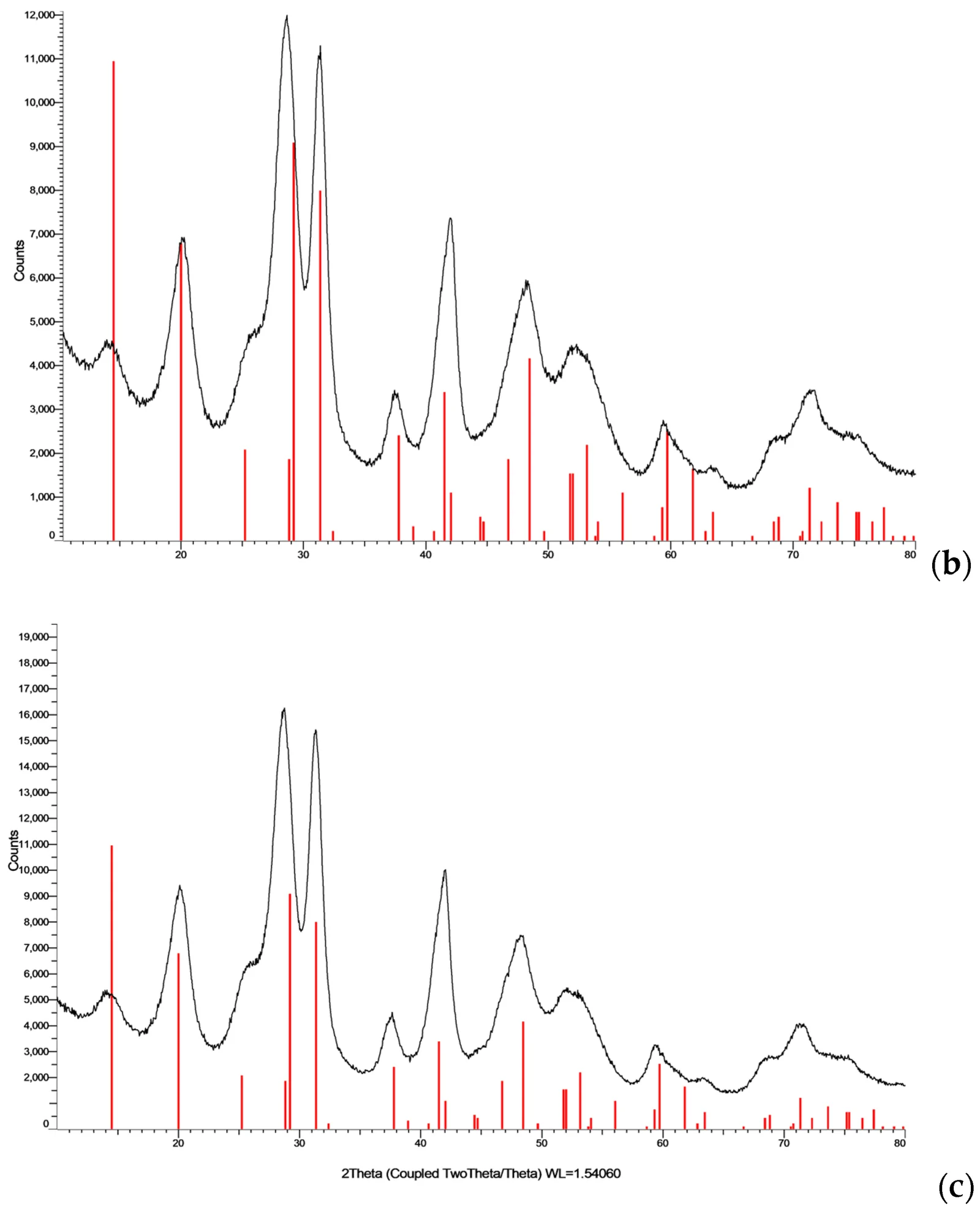
XRD patterns of annealed CePO_4_–CMCNa nanocomposite samples: (**a**) CMCNa 1.25; (**b**) CMCNa 0.625; and (**c**) CMCNa 0.625/6.25.

**Figure 4:**
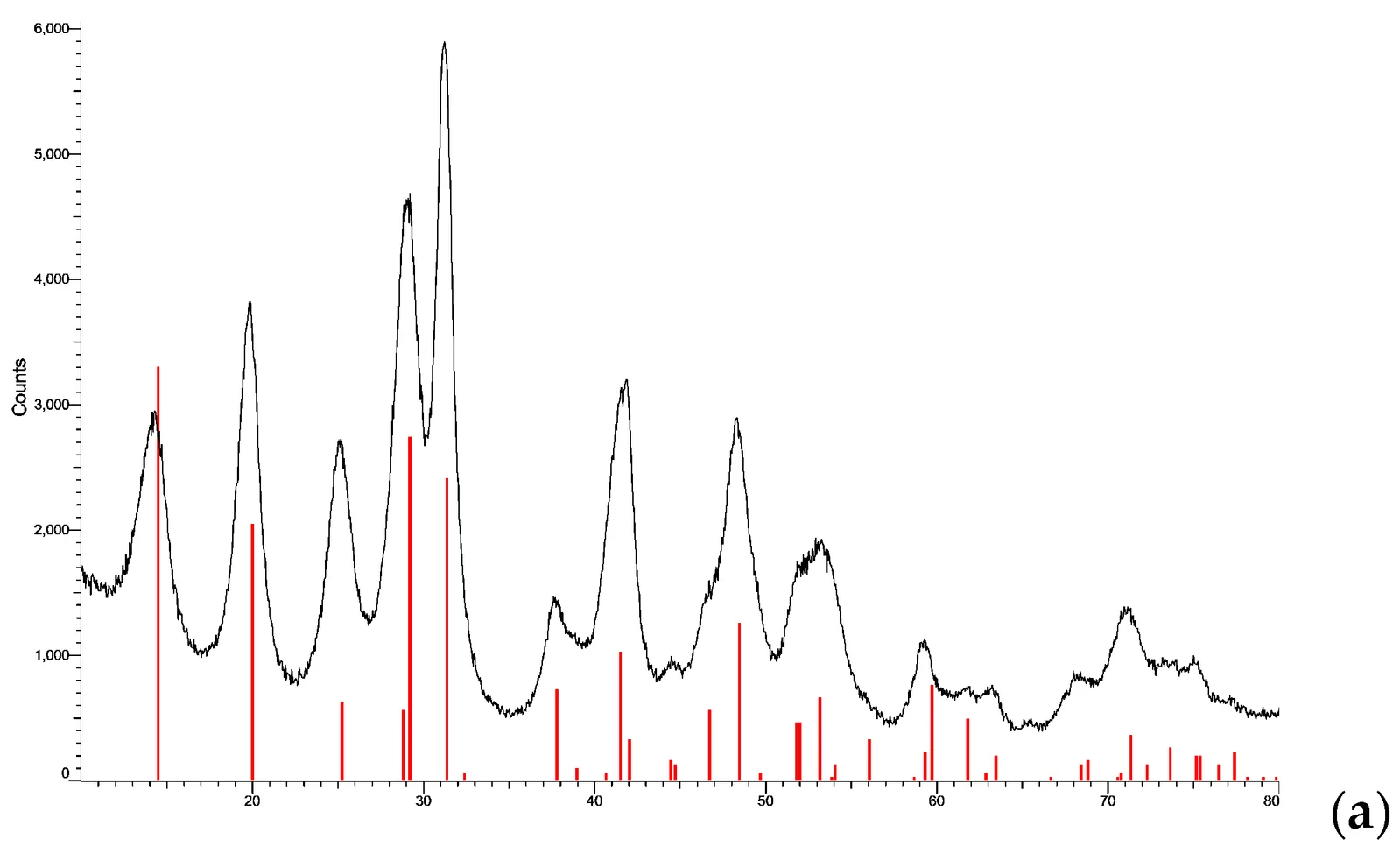

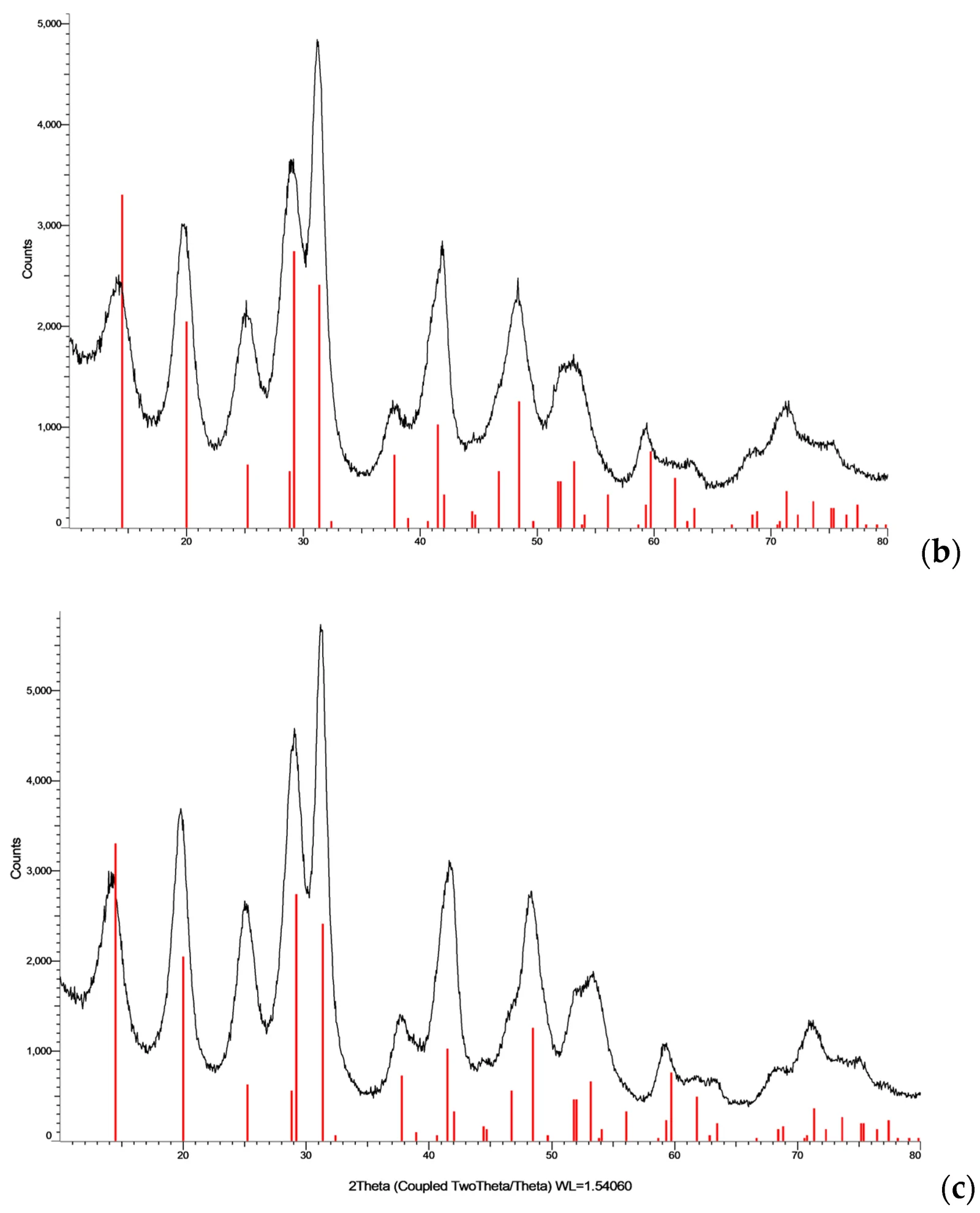
XRD patterns of cerium phosphate/polymer nanocomposites CePO_4_–Cellulose–Citrate: (**a**) MC + Cit 0.625/12.5; (**b**) CMC + Cit 0.625/12.5; and (**c**) CMCNa + Cit 0.625/12.5.

The diffraction peaks shown in Figure 2, Figure 3 and Figure 4 are in full agreement with the crystalline phase of cerium phosphate (CePO_4_). A comparison of the standard CePO_4_ peak widths (according to JCPDS/PDF card No. 34-1380, hexagonal phase with a rhabdophane-type structure) with the peaks widths obtained for the compounds under investigation shows that the peaks are approximately two to three times broader, both at the base and in the central region.

The pronounced broadening of the diffraction peaks is primarily attributed to the small size of the coherent scattering domains, reflecting the nanocrystalline nature of the crystallites. The slight broadening of the peak profiles and the elevated background are most likely associated with an amorphous polymer phase on the surface of the phosphate core. The initial, non-annealed CMCNa-basedsamples modified with metronidazole consisted of X-ray amorphous prior to drying and thermal treatment, indicating a high degree of structural disorder in the hydrated polymer networks, and the dried samples were the same single-phase cerium phosphate.

X-ray diffraction analysis of the CMCNa series samples annealed at 400 °C (Figure 3) also revealed the presence of a single cerium phosphate phase, despite the theoretically expected formation of cerium–sodium cerium–sodium phosphates. Compared with the as-prepared samples, the diffraction peaks became narrower, indicating an increase in crystallinity. In addition, an inversion in the relative intensities of the two main CePO_4_ reflections was observed, most likely resulting from texturing (preferred orientation) of the anisotropic crystallites induced by thermal treatment.

Analysis of the diffraction patterns of the powders synthesized from the initial solutions containing citric acid (Figure 4) confirmed the preservation of the target phase purity/ The incorporation of citric acid and metronidazole into the multicomponent systems also did not lead to a change in the phase composition of the cerium component or the formation of cerium citrate complexes.

### 3.2. Morphology, Particle Size, and Aggregation Stability of the Nanoparticles (TEM)

Comparison of TEM images showed the dependence of the morphology and size of the nanocomposites on the nature of the cellulose polymer used. In all cases, the resulting nanoparticles exhibited a pronounced anisotropic (rod-like or needle-like) morphology, which is probably due to the selective adsorption of polysaccharide macromolecules on specific crystallographic faces of the CePO_4_ nuclei, thereby restricting their lateral growth.

For all investigated systems, analysis of the selected area electron diffraction (SAED) ring patterns confirmed their complete correspondence to the hexagonal CePO_4_ phase.

**Figure 5:**
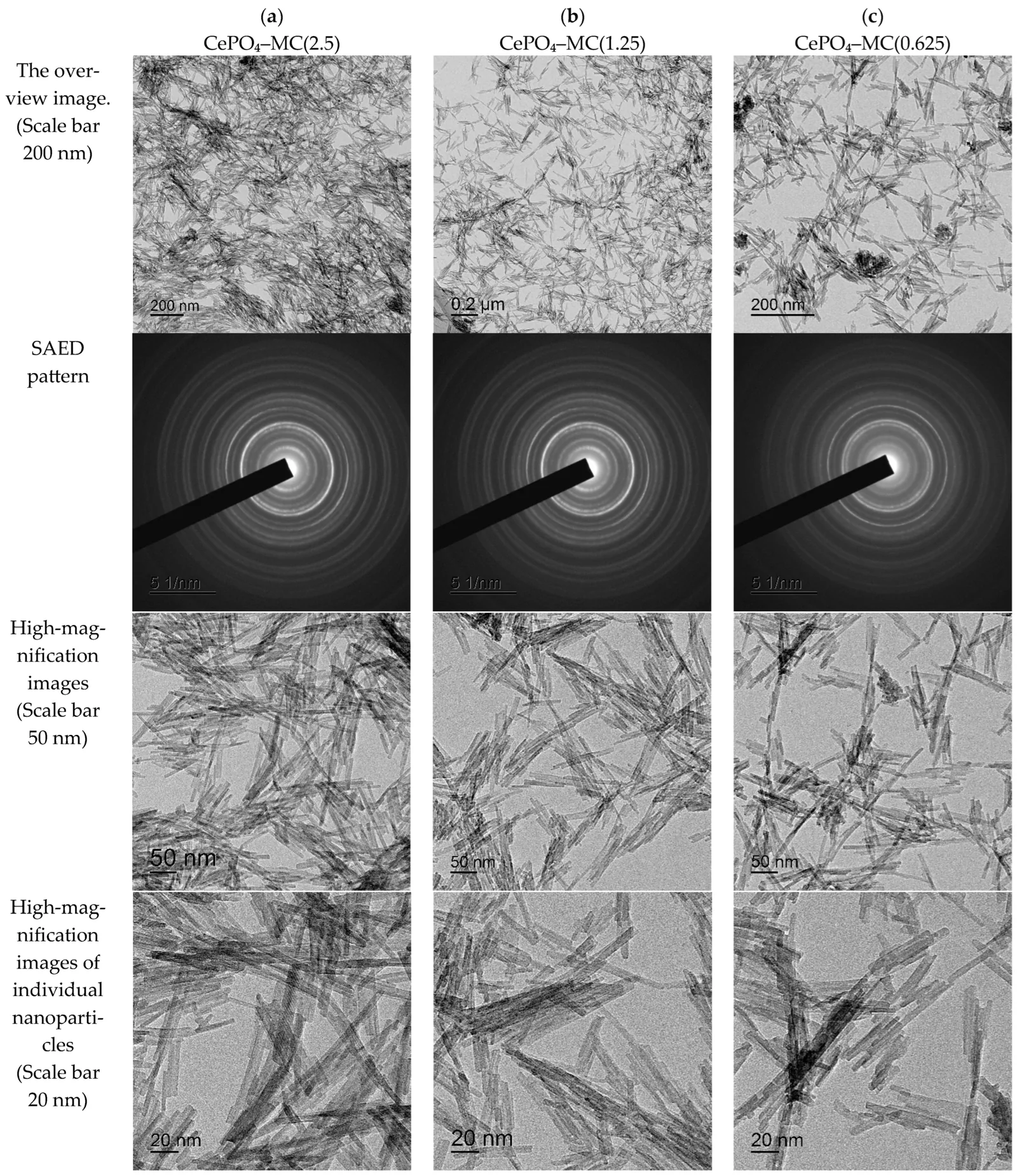
TEM images of nanocomposite particles in CePO_4_-methylcellulose (MC) samples: (**a**) MC 2.5, (**b**) MC 1.25, and (**c**) MC 0.625.

Figure 5 shows TEM images of the nanocomposite particles in cerium(III) phosphate samples modified with methylcellulose (MC). The samples are moderately agglomerated and consist of loosely packed, randomly distributed aggregates of fine particles. The nanoparticles exhibit a rod-like morphology, with widths ranging from 3 to 6 nm and lengths varying from 100 to 200 nm. High-resolution images clearly reveal the crystal lattice of the individual nanorods. Variation of the MC concentration (2.5, 1.25, and 0.625 g/L) does not significantly affect the geometry of the individual crystallites but acts as a regulator of their secondary aggregation.

**Figure 6:**
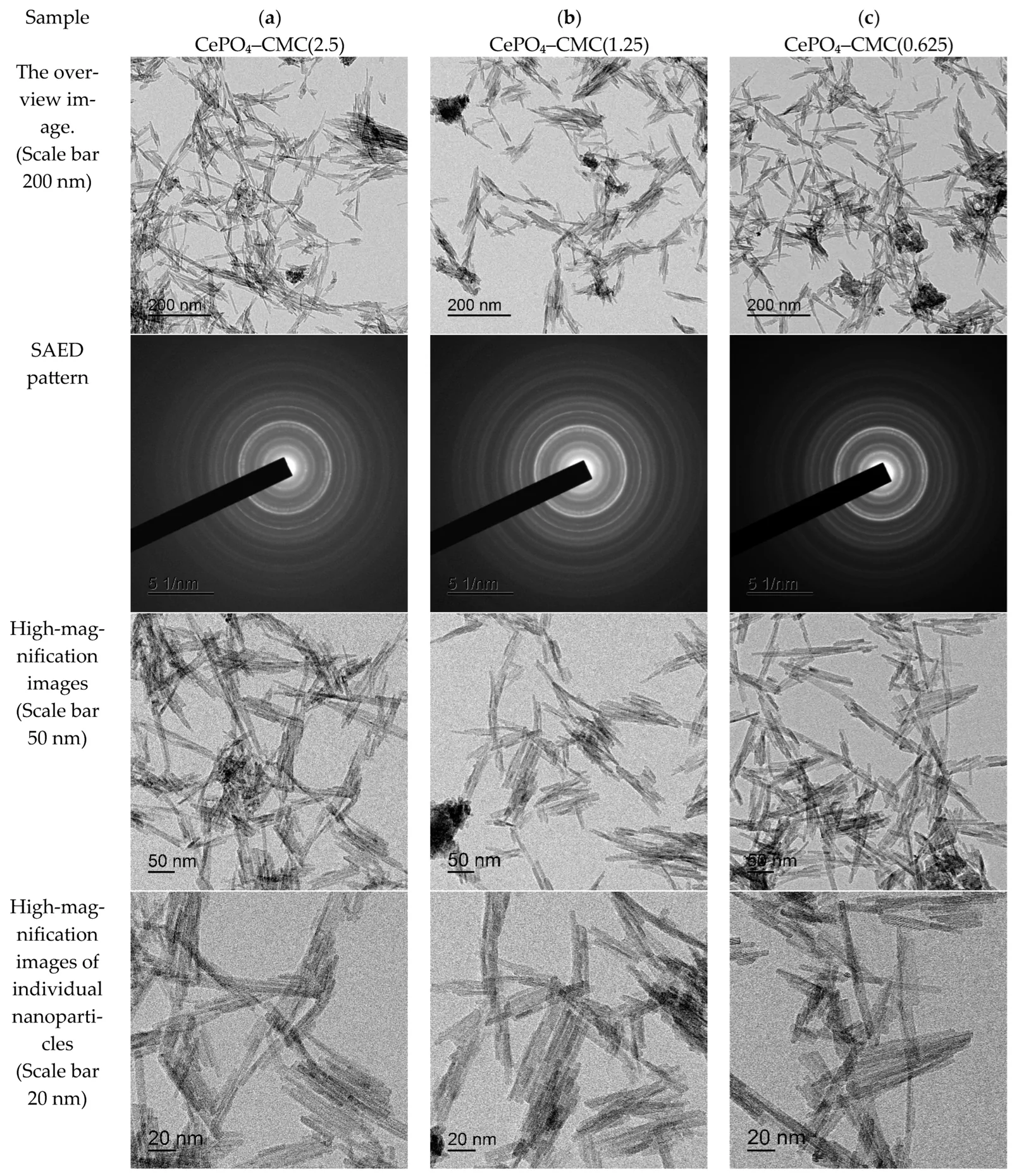
TEM images of nanocomposite particles in CePO_4_/carboxymethyl cellulose (CMC) samples: (**a**) CMC 2.5, (**b**) CMC 1.25, and (**c**) CMC 0.625.

The use of CMC as a modifier (Figure 6) results in the formation of nanoparticles with average dimensions slightly smaller than those obtained using nonionic MC. We assume that this is attributed to the stronger polyelectrolyte interaction between the anionic carboxylate groups of the polymer (-COO^−^-) and (Ce^3+^) ions during the nucleation stage.

**Figure 7:**
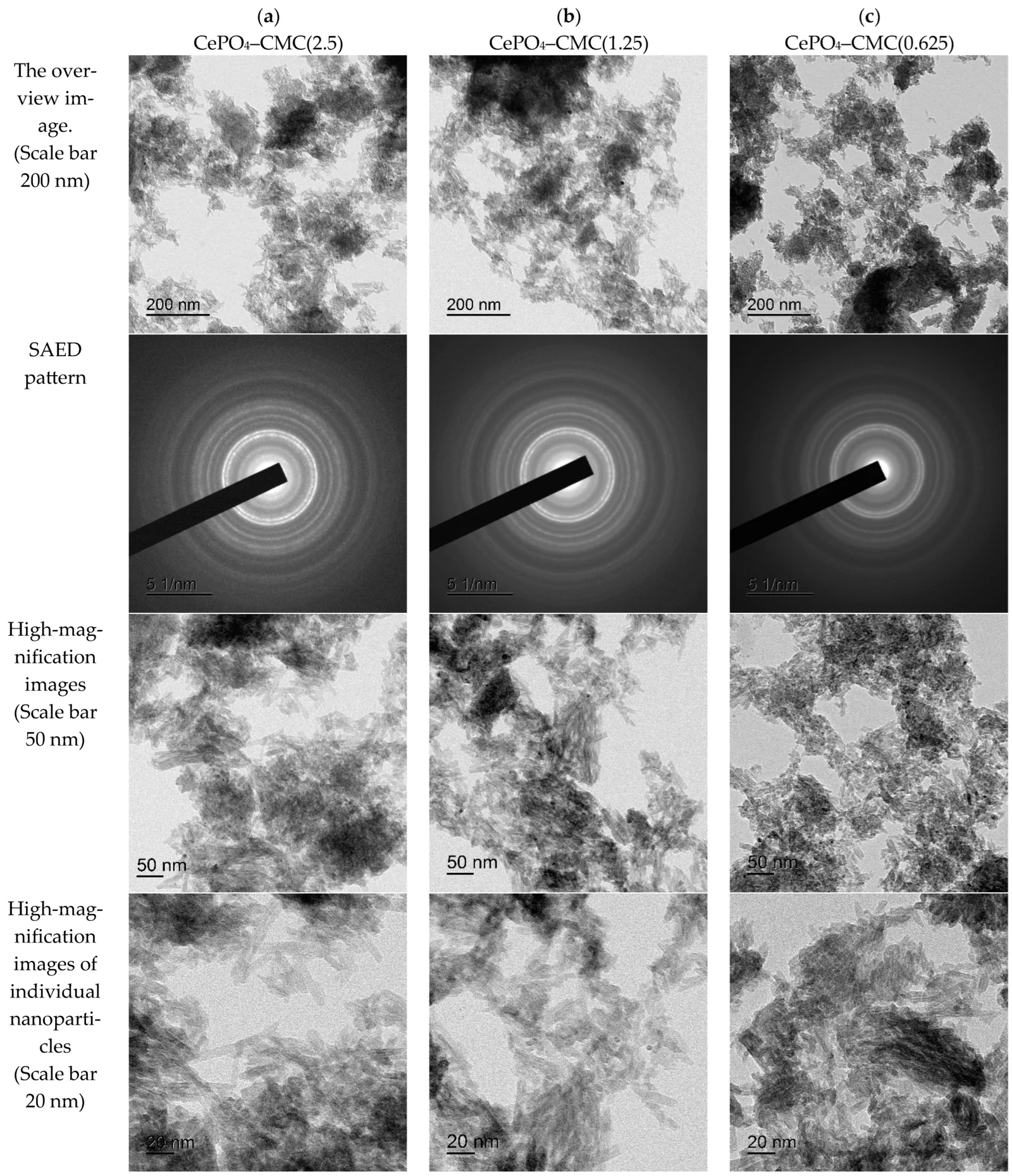
TEM images of annealed nanocomposite particles in CePO_4_/carboxymethyl cellulose (CMC) samples: (**a**) CMC 2.5, (**b**) CMC 1.25, (**c**) CMC 0.625.

For the samples containing CMC, MC and CMCNa, this temperature is insufficient; calcination at 400 °C leads to significant burnout of the organic component and slightly changes the particle morphology (Figure 7). After calcination, particle aggregation increases significantly, and conglomerates with sizes of 200 nm and larger are formed, consisting of individual crystallites approximately 5 nm in width and with a maximum length of 80 nm.

**Figure 8:**
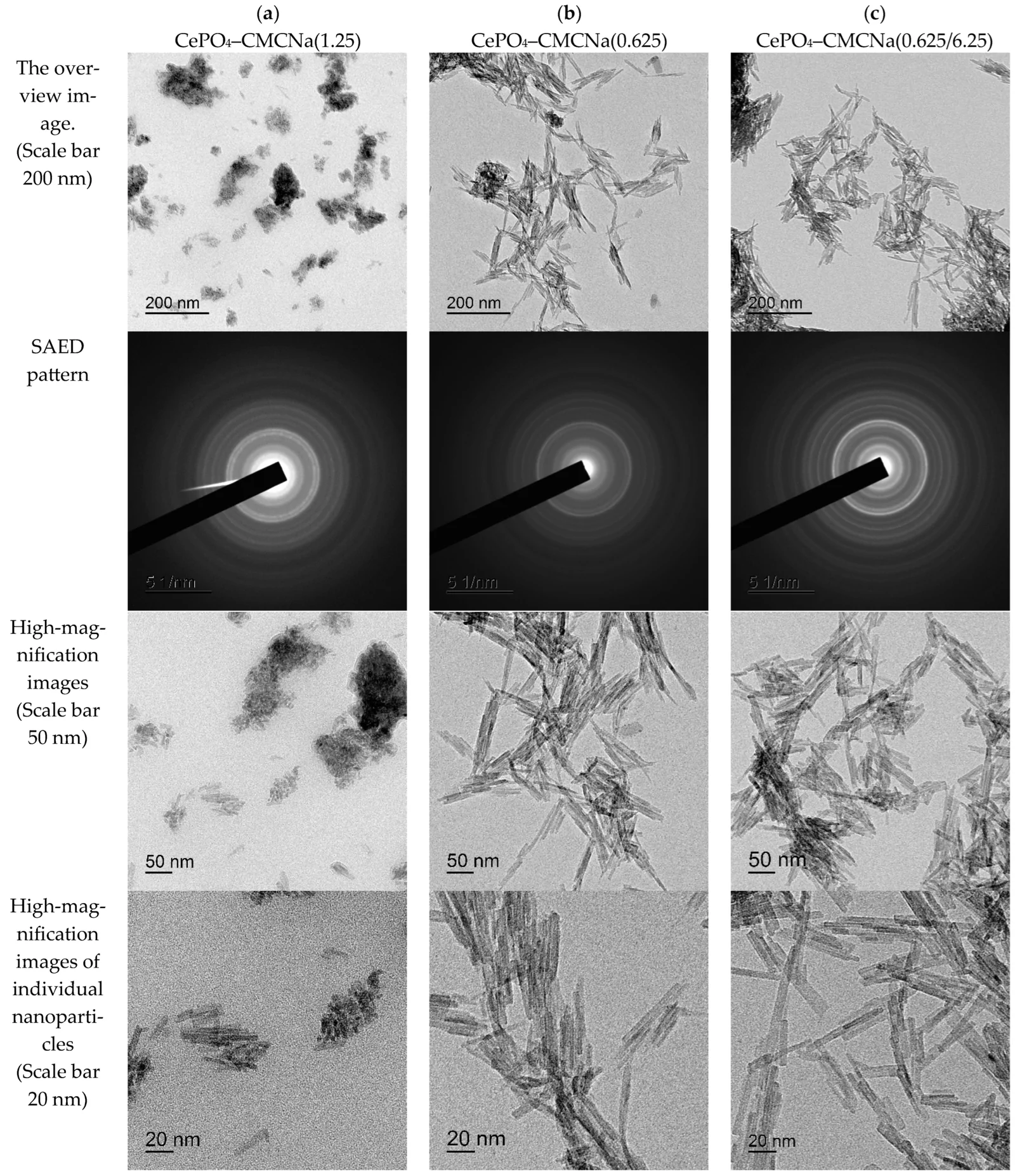
TEM images of nanocomposite particles in CePO_4_/sodium carboxymethyl cellulose (CMCNa) samples: (**a**) CMCNa 1.25, (**b**) CMCNa 0.625, and (**c**) CMCNa 0.625/6.25.

Figure 8 shows TEM images of nanocomposite particles in non-annealed cerium(III) phosphate samples modified with CMCNa. In all samples, agglomerates with sizes of approximately 200 nm or larger were observed, consisting of randomly distributed clusters of fine composite particles. The nanoparticles exhibit an anisotropic morphology, with widths remaining nearly constant at approximately 3–5 nm, whereas their lengths vary over a wide range. Individual particles reach lengths of up to 100 nm.

The nanoparticles synthesized from CMCNa-containing solutions exhibit characteristics similar to those of the CMC-derived samples. The obtained results demonstrate that variations in the concentrations of the initial solutions influence both the particle dimensions and the degree of agglomeration. A higher CMCNa concentration concentration increases the viscosity of the reaction medium, which prevents the linear growth of nanocomposites. Conversely, a decrease in the concentration of the cerium component results in a slight reduction in both the width and the length of the cerium phosphate nanoparticles.

**Figure 9:**
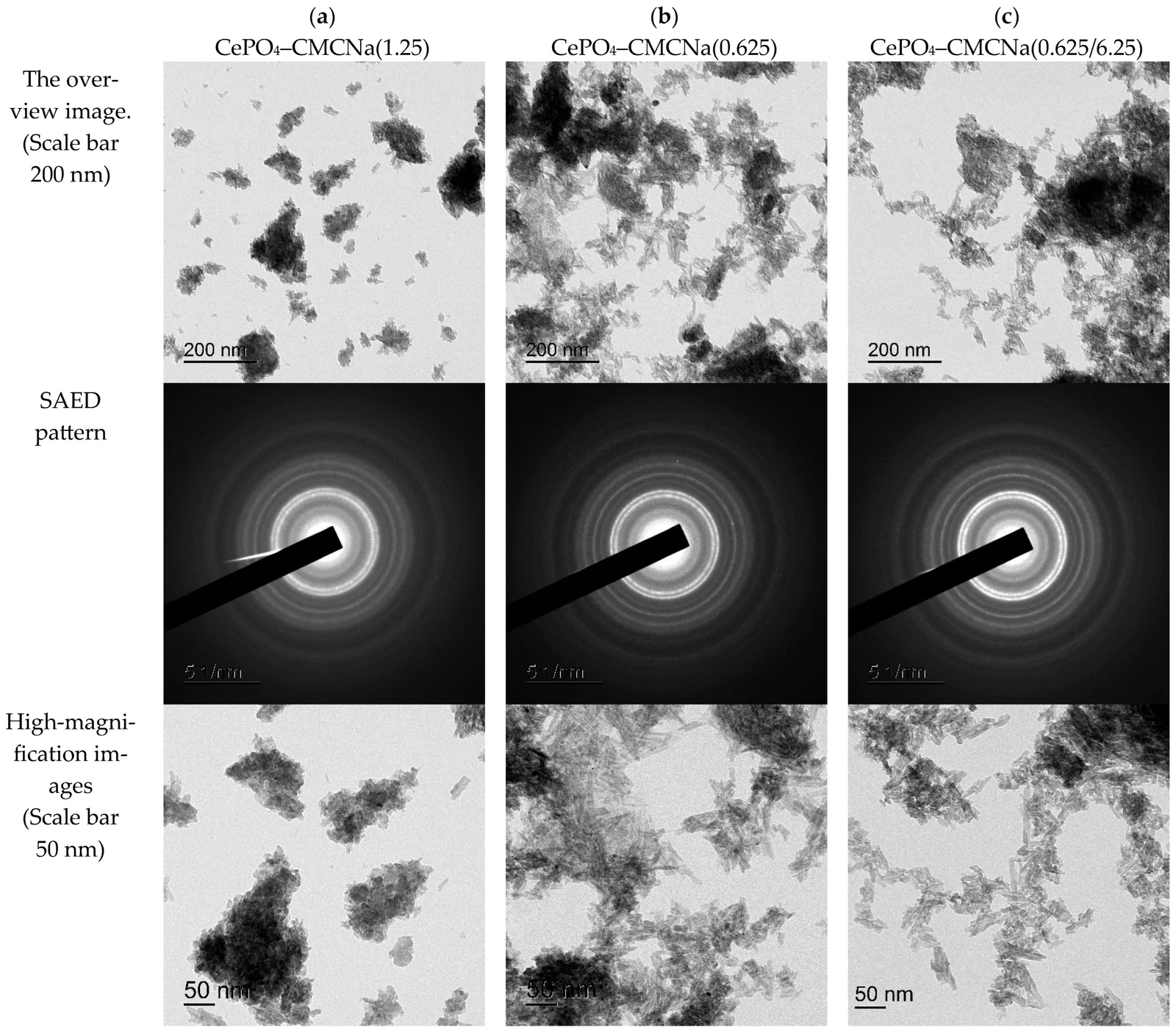

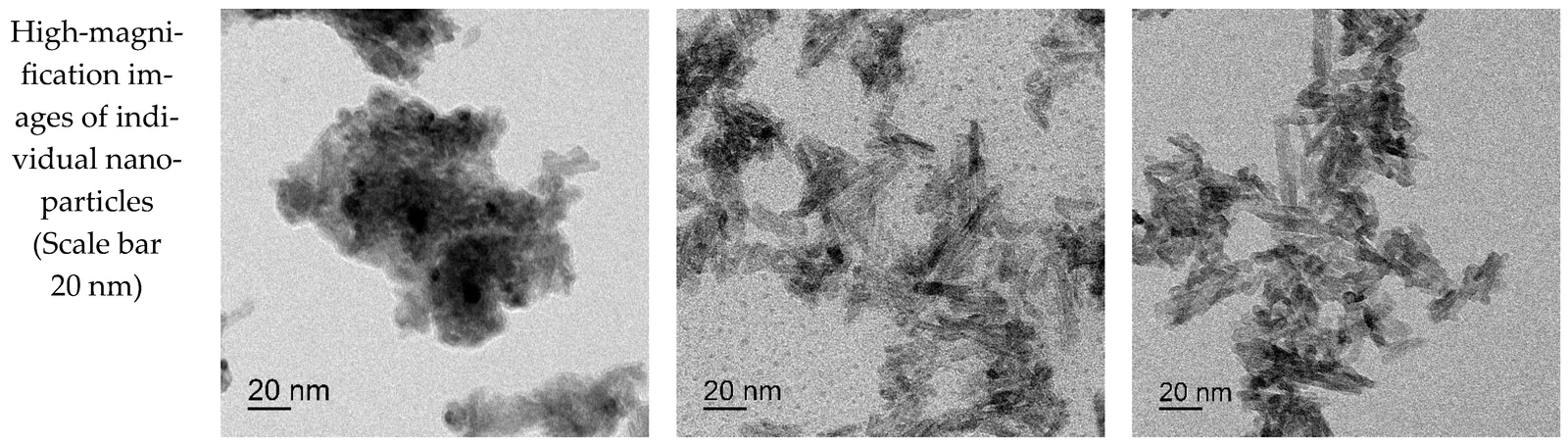
TEM images of annealed nanocomposite particles in CePO_4_/sodium carboxymethyl cellulose (CMCNa) samples: (**a**) CMCNa 1.25, (**b**) CMCNa 0.625, and (**c**) CMCNa 0.625/6.25.

To further investigate the effect of annealing on the dimensions of the CMC-CePO_4_ nanocomposites, the obtained powders were also annealed at 400 °C. TEM images acquired after annealing (Figure 9) show that all samples contain agglomerates with sizes of approximately 200 nm or larger, consisting of randomly distributed clusters of fine composite particles. The particles forming these agglomerates retain an anisotropic morphology, with widths remaining nearly constant at approximately 3–5 nm, whereas their lengths range from 15 to 30 nm.

The annealed CePO4 nanoparticles, obtained in the presence of any form of the cellulose matrix, have a shorter length than the particles in the corresponding non-annealed samples. This indirectly confirms the assumption that, in all cases of phosphate synthesis from solutions containing both CMC and CMCNa, crystallization of fine phosphate particles occurs within the space of the cellulose matrix, between the cellulose fibers. Removal of cellulose during calcination does not lead to linear growth of cerium phosphate crystals but increases their agglomeration.

**Figure 10:**
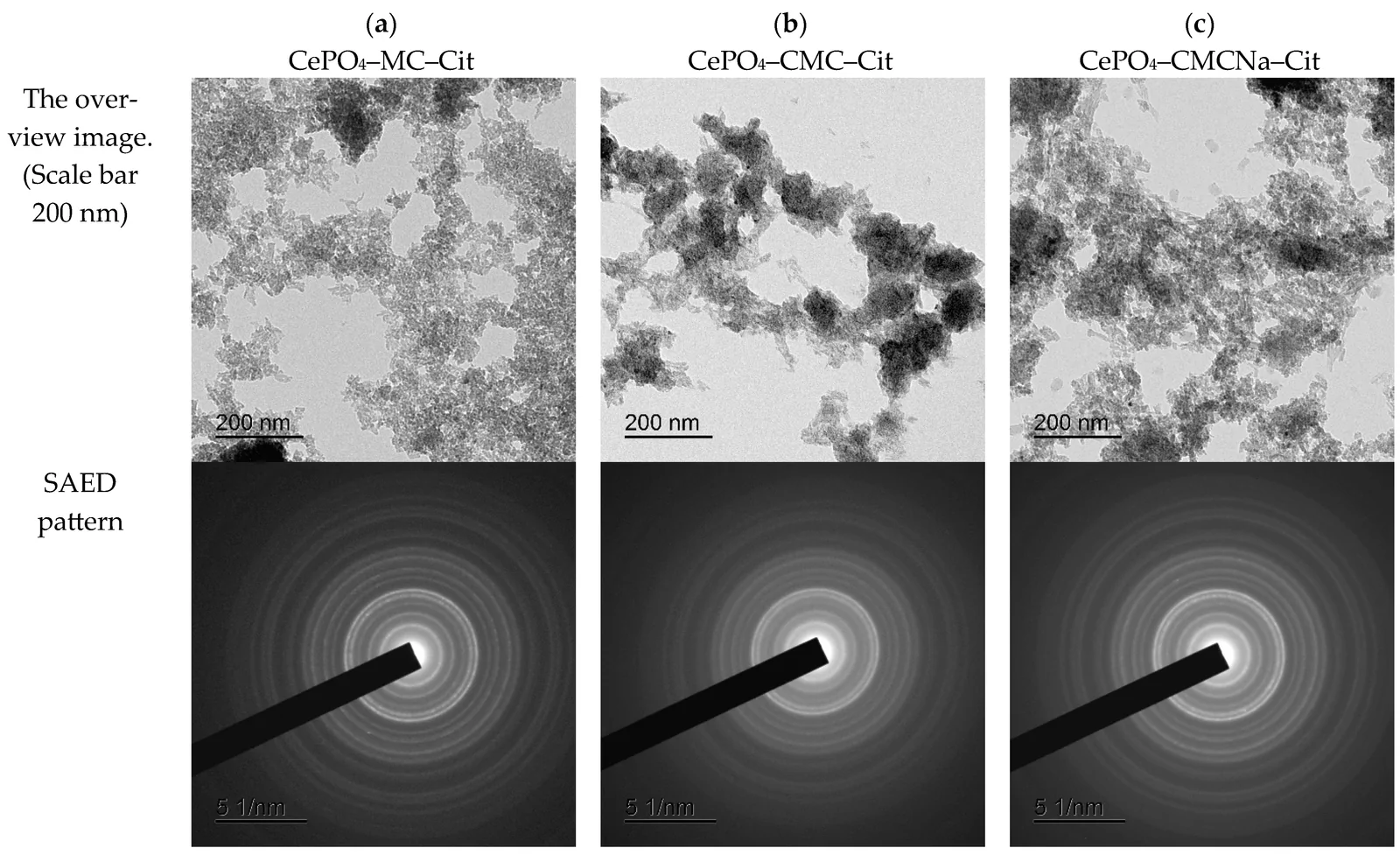

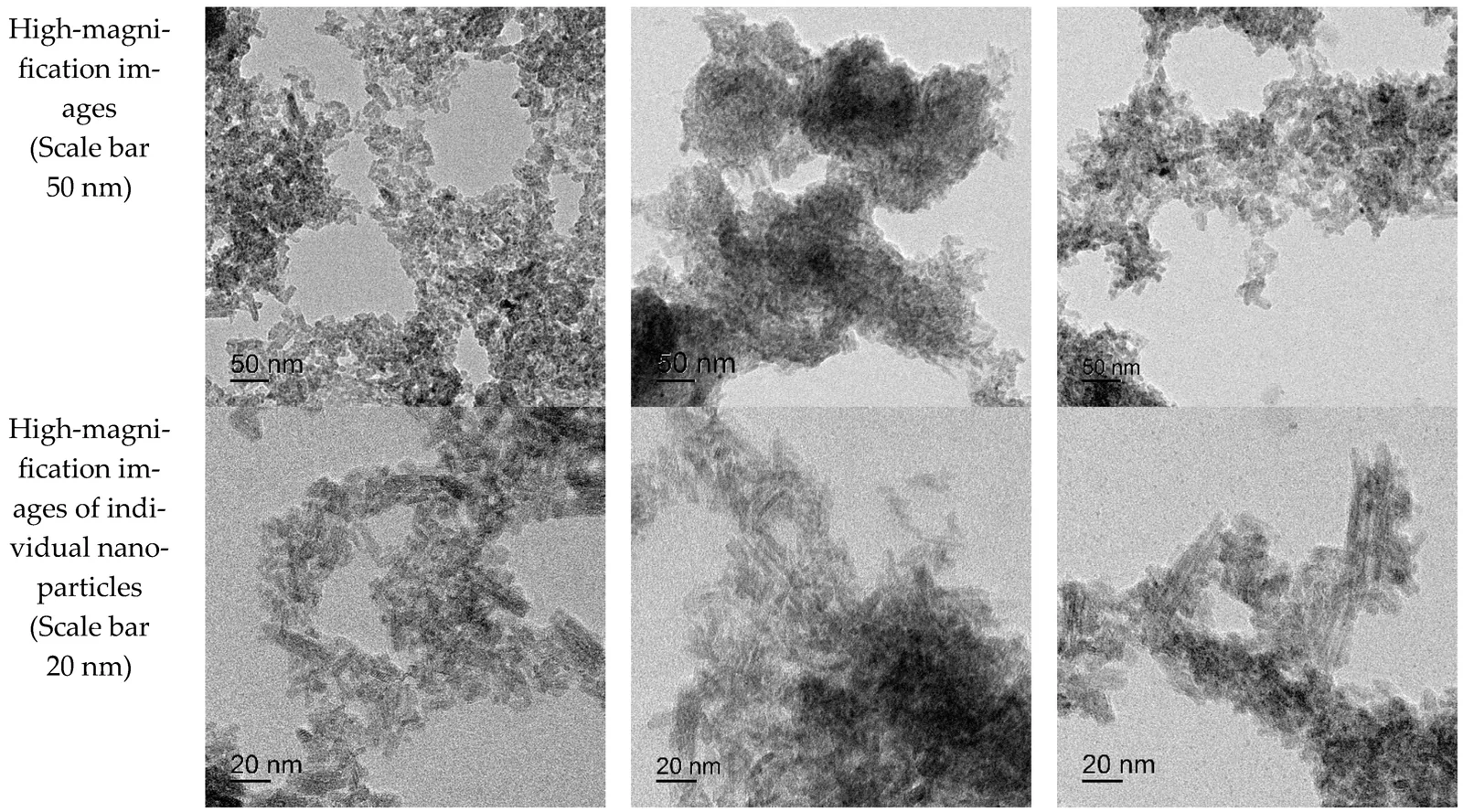
TEM images of nanocomposite particles in (**a**) CePO_4_ + MC + Citrate, (**b**) CePO_4_ + CMC + Citrate, and (**c**) CePO_4_ + CMCNa + Citrate samples.

TEM micrographs of the samples modified with citric acid (Figure 10) confirm its effect on limiting nanoparticle growth: their length decreases by tens of times (some crystallites are only 5–7 nm long with a width of 2–5 nm; individual rods reach 25 nm), which once again confirms our observations regarding the role of citric acid as a limiter of the linear growth of crystals (compared with crystals obtained in aqueous [43,65] and cellulose-containing media [64]). Similar results have also been reported in other studies [66,67,68].

### 3.3. Investigation of Intermolecular Interactions (FTIR Spectroscopy)

**Figure 11:**
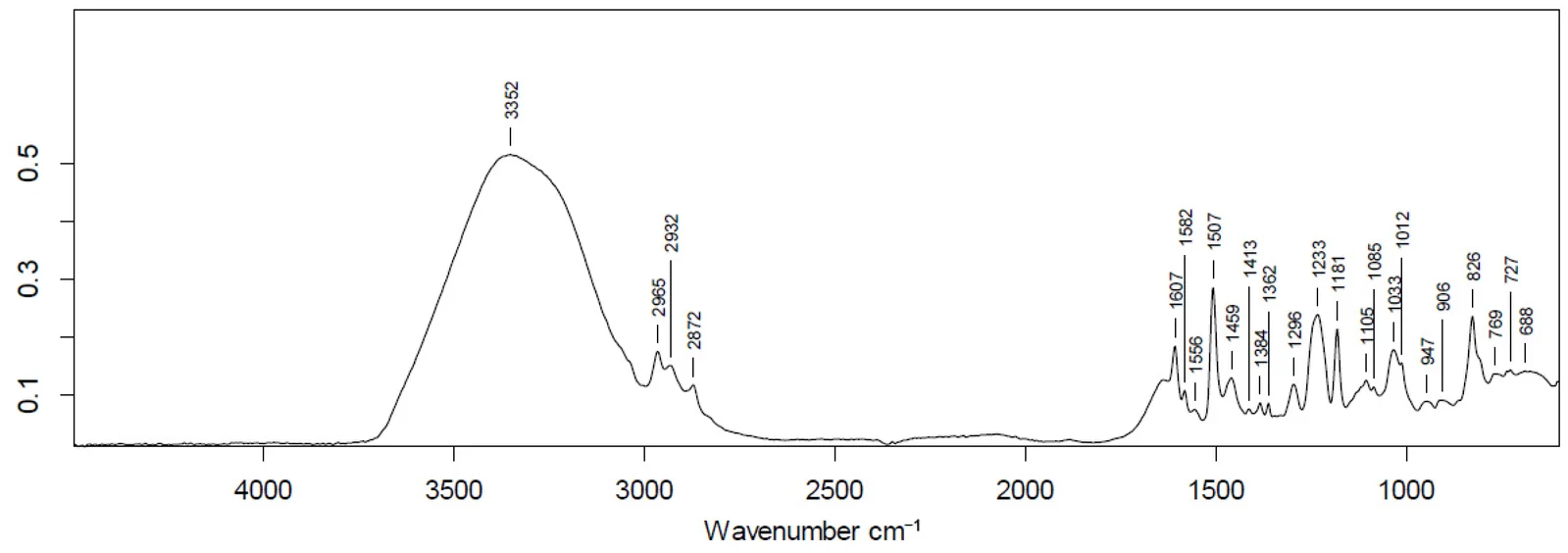
FTIR spectrum of the CePO_4_–citrate–CMCNa–metronidazole composite.

**Figure 12:**
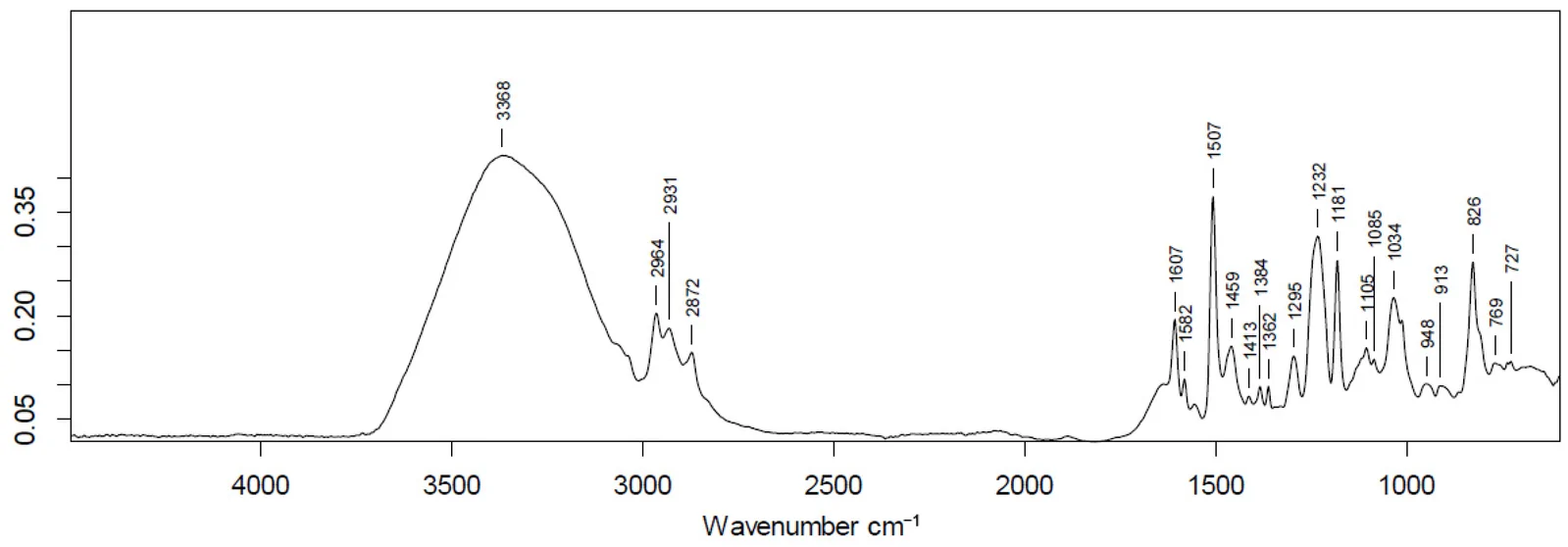
FTIR spectrum of the CePO_4_–MC composite.

**Figure 13:**
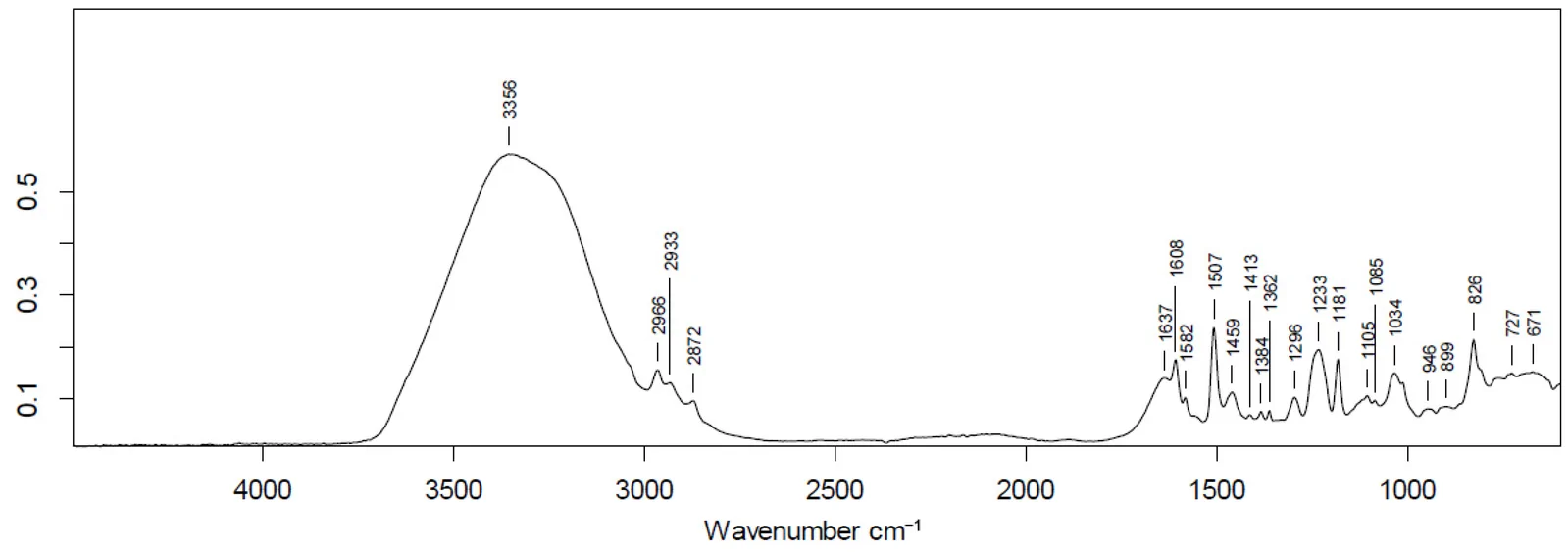
FTIR spectrum of the CePO_4_–MC–citric acid composite.

Based on the FTIR spectra of the starting components and the obtained composites (Figure 11, Figure 12 and Figure 13 and Table 1, Table 2 and Table 3 with assignments of the FTIR absorption bands), several conclusions can be drawn regarding the coordination mode, intermolecular interactions, and the successful formation of the hybrid materials.

The spectrum of free citric acid exhibits intense absorption bands corresponding to the stretching vibrations of carbonyl (C=O) groups in the 1722–1686 cm^−1^ region. Sodium carboxymethyl cellulose (CMCNa) displays a band at 1588 cm^−1^ assigned to the asymmetric stretching vibrations of the carboxylate anion (COO^−^).

In the spectra of all composites containing CMC and citric acid, the high-frequency absorption bands of free carboxylic groups (1722–1686 cm^−1^) disappear completely. Instead, an intense band appears at 1607–1608 cm^−1^. This indicates complete deprotonation of the carboxyl groups of citric acid and CMCNa, as well as their chelation or bridging coordination with Ce^3+^ ions. The shift of the COO^−^ absorption band to approximately 1607 cm^−1^ is characteristic of metal–ligand coordination.

For nonionic methylcellulose (MC), which contains no carboxyl groups, the band at approximately 1607 cm^−1^ is assigned to the bending vibrations of coordinated (bound) water molecules. Analysis of the spectra of the binary CePO_4_–MC system shows that the polymer macromolecules are retained on the surface of the inorganic phase predominantly through an extended network of weak hydrogen bonds. This is evidenced by the broadening and high-wavenumber shift of the O–H stretching band from 3241–3173 cm^−1^ in the initial polymers to 3352–3368 cm^−1^ in the composites. Instead of strong polymer–polymer interactions, an interconnected hydrogen-bonding network is formed between the cellulose hydroxyl groups, water molecules, and phosphate centers.

The band at 1507 cm^−1^ is observed in all three materials. Most probably, it corresponds to the deformation vibrations of bound water molecules. The overtone of the deformation vibrations of water molecules is usually observed at approximately 1630–1640 cm^−1^. However, for water molecules that have formed a system of strong hydrogen bonds with the hydroxyl (-OH) and methoxy (-OCH_3_) groups of the cellulose framework, this band may exhibit a pronounced shift to the lower-frequency region (1500–1520 cm^−1^).

The three bands (1295, 1232 and 1181 cm^−1^) can be assigned to vibrations of cellulose fragments of the compounds. Thus, the bands observed at 1295 and 1232 cm^−1^ correspond to the in-plane deformation vibrations of hydroxyl groups and the stretching vibrations of pyranose rings in polysaccharides, whereas the band at 1181 cm^−1^ is attributed to the asymmetric stretching vibrations of the C-O-C glycosidic bond connecting glucose units.

Band at 826 cm^−1^. This band is assigned either to deformation vibrations of Ce–O bonds or to deformation vibrations of coordinated organic molecules, confirming the formation of a stable coordination framework.

All composite samples exhibit a broad, complex absorption band in the 1033–1034 cm^−1^ region, which includes the asymmetric stretching vibrations of P–O bonds in phosphate anions (PO_4_^3−^) together with vibrations involving Ce–O bonds.

These observations confirm the successful precipitation and formation of the crystalline or amorphous cerium orthophosphate (CePO_4_) phase, which is firmly integrated into the polymer matrix (MC/CMCNa).

The -OH stretching bands of the initial polymers (MC: 3241 cm^−1^; CMCNa: 3173 cm^−1^) become broader and shift toward higher wavenumbers (3352–3368 cm^−1^) in the composite materials.

This indicates a rearrangement of the hydrogen-bonding network. Instead of strong intermolecular polymer–polymer interactions, an extended hydrogen-bonding network is formed between the hydroxyl groups of methylcellulose or CMC, coordinated water molecules, and phosphate centers.

Analysis of the IR spectra of the two-component systems (CePO_4_–MC and CePO_4_–CMC) showed the absence of characteristic bands that could indicate the formation of strong coordination or covalent bonds between the functional groups of cellulose ethers and defects in the cerium phosphate lattice. The polymer macromolecules are retained on the surface of the inorganic nanorods by weak hydrogen bonds. The main role of MC and CMC is limited to rheological control of the precipitation medium and spatial restriction of ion migration.

In the spectra of the samples synthesized with the addition of citric acid, a shift of the absorption bands corresponding to the skeletal stretching vibrations of the C–C bonds of citric acid was observed. This shift confirms the formation of a stable CePO_4_–citric acid nanocomposite due to the coordination of carboxylate groups to surface cerium ions. At the same time, no direct spectral evidence of interaction between the cellulose matrix (MC/CMC) and citric acid was detected.

The introduction of metronidazole into the system complicates the IR spectral profile in the 3100–3600 cm^−1^ region because of the multiple superposition and overlap of intense broad stretching vibration bands of the hydroxyl groups (OH^−^) of residual water, cellulose units and citric acid, which somewhat complicates their assignment. However, the combination of the obtained data, preservation of the phase according to XRD, and the presence of the combined organic and inorganic bands in the IR spectra indirectly indicate the successful incorporation of metronidazole into the nanocomposite.

## 4. Discussion

In recent decades, nanomaterials have become transformative tools in pharmacotherapy and biomedicine, making it possible to overcome the key limitations of conventional dosage forms, such as low bioavailability, insufficient solubility, rapid systemic clearance, and pronounced side effects [1,2,3,4,5]. One of the most promising directions is the development of new nanomaterials based on rare earth elements possessing regenerative properties and pronounced enzyme-mimetic activity (oxidase-, peroxidase-, catalase-, and phosphatase-like) [5,6,7], especially based on cerium compound nanozymes capable of effectively destroying bacterial biofilms and protecting cellular structures from destructive oxidative stress, thereby creating optimal conditions for tissue regeneration and wound healing [8,9,28,38,39,40,41,42,50]. However, while the beneficial biological effects of cerium oxide nanoparticles have been studied for a long time by numerous researchers from various countries, research into the potential applications of cerium phosphate nanoparticles in biomedical fields is relatively new [64,65] Water-soluble cellulose derivatives—methylcellulose (MC) and carboxymethylcellulose (CMC)—have been successfully used as biopolymer matrices for stabilizing and directing the formation of these nanostructures [20,21,22,23,24,25,26]. Acting as rheological modifiers and stabilizers, these cellulose ethers not only promote the preferential precipitation of small, isolated crystallites but also provide the moisture retention required in the wound area [4,31].

The present study is a continuation of a series of studies devoted to the development of a new generation of pharmaceuticals based on redox-active cerium oxide and cerium phosphate nanoparticles in biopolymer matrices with antimicrobial activity for accelerating wound regeneration [10,38,40,43,50,63,64,65]. Initially, we studied the possibility of synthesizing core-less cerium phosphate nanoparticles, in particular, trivalent cerium phosphate [43]; in our latest study, we investigated the possibility of creating a CePO4-citrate nanocomposite by selecting optimal dosages of citric acid [65]. Unlike our previous studies, in this work we have, for the first time, carried out a comprehensive series of syntheses of nanocomposites based on cerium(III) orthophosphate stabilized with various forms of cellulose (MC, CMC, CMCNa) at different concentrations with the addition of different concentrations of citric acid. Characterization of the synthesized novel hybrid nanocomposites made it possible to reveal the regularities of the influence of the nature and concentration of various forms of cellulose and tricarboxylic acids on the morphology, dispersity, and aggregative stability of the nanoparticles and, ultimately, to select the optimal nanocomposite formulation and demonstrate the possibility of metronidazole encapsulation in the CePO_4_–citrate–cellulose system developed by us.

The obtained results confirm the working hypothesis regarding the possibility of directed regulation of the morphology of a nanosized cerium(III) orthophosphate composite by varying the nature of the biopolymer matrix and the addition of organic components. The formation of an exclusively hexagonal phase CePO_4_ (rhabdophane structural type), detected by X-ray diffraction in all synthesized samples, is fully consistent with the regularities of low-temperature precipitation of rare earth phosphates. The high dispersity of the phosphate phase is retained even after calcination at 400 °C, whereas, in the absence of the polymer matrix, nanoparticle coarsening occurs.

Analysis of the transmission electron microscopy data makes it possible to propose a mechanism for the influence of organic polymer additives on the geometry and habit of the nanoparticles. The constant width of the nanocrystals (3–6 nm) suggests that the growth of the lateral faces is limited by steric factors. The decrease in the length of the nanorods from 200 to 15 nm in the presence of citric acid confirms its role as a selective regulator of linear dimensions. Citric acid exhibits a high affinity for cerium ions—the overall stability constants (logβ) for the citrate complexes of rare earth elements formed in aqueous solutions range from 9.1 to 11.2, depending on pH and ionic strength (taking into account both average monocomplexes and protonated species of the type ([M(HCit)]^+^) [69,70]. We may assume that citric acid molecules are selectively coordinated on the terminal faces of the growing crystals, thereby restricting their further growth.

The difference in the effect of nonionic MC and the anionic forms CMC and CMCNa on the size of the resulting particles is apparently explained by the different surface charge density of the polymer chains and the contribution of electrostatic interactions. It follows that changing the composition of the cellulose matrix will alter the morphology of the nanocomposites.

The observed shift of the characteristic absorption bands of the functional groups in the IR spectra indicates that the cellulose polymer chains are retained on the surface CePO_4_ predominantly through an extensive network of weak hydrogen bonds. In contrast, the carboxylate groups of citric acid exhibit a pronounced shift characteristic of the formation of coordination bonds with surface Ce^3+^ centers.

The addition of metronidazole demonstrates the possibility of creating multifunctional materials for regenerative medicine. The combination of the bacteriostatic action of metronidazole and the enzyme-mimetic properties of cerium orthophosphate would make it possible to simultaneously address the suppression of pathogenic microflora and the reduction of excessive oxidative stress in the wound area.

### Limitations and Prospects

The main objective of this study was to investigate the effect of various forms of cellulose on the morphology of the nanoscale citrate complex of cerium phosphate, to assess its stability, and to verify the possibility of incorporating (encapsulating) the medicinal drug into the structure of the nanocomposite. This article does not discuss the dosage of the drug, but only the potential possibility of creating a hybrid nanocomposite. Our next studies will focus on the dosages and effectiveness of nanocomposites for biomedical applications. In addition, our future studies will be aimed at obtaining cellulose films and hydrogels containing cerium nanocompounds (phosphate or oxide), a complexing agent (carboxylic acid), and an affordable antibiotic (including metronidazole). We hope that the developed nanocomposites will have good prospects for application in biomedicine as prolonged wound-healing and antibacterial agents.

## 5. Conclusions

1. A method for the synthesis of cerium(III) phosphate nanocomposite powders modified with various water-soluble forms of cellulose and citric acid has been developed and optimized, allowing control of the morphology and dispersity of the resulting systems.
2. The influence of the nature and concentration of cellulose ethers on the properties of the nanoparticles has been established: nonionic methylcellulose promotes the formation of elongated rod-like particles (100–200 nm) with moderate loose agglomeration, whereas anionic carboxymethylcellulose (CMC) and its sodium salt form shorter crystallites prone to the formation of bulky and loose nanoaggregates.
3. The positive effect of citric acid on limiting the linear dimensions of cerium(III) phosphate crystallites in the presence of a cellulose matrix has been demonstrated.
4. X-ray diffraction confirmed the formation of single-phase cerium(III) orthophosphate in all experimental series. This structure is retained after calcination at 400 °C, which is apparently due to the shielding effect of the polymer matrix.
5. According to transmission electron microscopy data, the individual nanoparticles exhibit pronounced anisotropy with a fixed width of 3–6 nm and a length ranging from 15 to 200 nm, depending on the rheological parameters of the medium and the nature of the coordination of the stabilizing agents.
6. Experimental samples of cerium phosphate nanocomposites modified with metronidazole were obtained for the first time during the synthesis process. It appears that the stability of the composite is ensured by hydrogen bonding between cellulose molecules and the coordination of citric acid with surface cerium ions.

## Figures and Tables

**Table 1:** Assignment of the FTIR absorption bands of the CePO_4_–citric acid–CMCNa–metronidazole composite.

3352, 2965	O–H stretching vibrations of hydrogen-bonded hydroxyl groups
2932	C–H stretching vibrations in glucose rings
1607	C=O stretching vibrations (carbonyl group)
1507	Deformational vibrations of water molecules
1459	CH_2_ and CH_3_ bending vibrations
1296, 1233, 1181	C–C skeletal stretching vibrations (citric acid, metronidazole)
1033	Skeletal vibration of the –CH_2_– group in the ring Asymmetric P–O stretching vibrations in phosphate anions Ce–O stretching vibrations P–O–Alk stretching vibrations
826	Deformational vibrations of Ce-O metal-oxygen bonds

**Table 2:** Assignment of the FTIR absorption bands of the CePO_4_–MC composite.

3368	O–H stretching vibrations of hydrogen-bonded hydroxyl groups
2964	C–H stretching vibrations in glucose rings
2931	C–H stretching vibrations in glucose rings
2872	C–H stretching vibrations in glucose rings
1607	O–H overtone vibration
1582	Breathing stretching vibrations of the carbon skeleton in the ring
1507	Deformational vibrations of water molecules
1459	CH_2_ and CH_3_ bending vibrations
1295, 1232	Deformational vibrations of hydroxyl groups δ(O-H), valence vibrations of pyranose rings
1181	Asymmetric valence vibrations of the C-O-C glycosidic bond
1034	Skeletal vibration of the –CH_2_– group in the ring Asymmetric P–O stretching vibrations in phosphate anions Ce–O stretching vibrations P–O–Alk stretching vibrations
826	Deformational vibrations of Ce-O metal-oxygen bonds

**Table 3:** Assignment of the FTIR absorption bands of the CePO_4_–MC–citric acid composite.

3356	O–H stretching vibrations of hydrogen-bonded hydroxyl groups
2966	C–H stretching vibrations in glucose rings
2933	C–H stretching vibrations in glucose rings
1608	C=O stretching vibrations (carbonyl group); O–H overtone band
1507	Deformational vibrations of water molecules
1459	CH_2_ and CH_3_ bending vibrations
1296, 1233	Deformational vibrations of hydroxyl groups δ(O-H), valence vibrations of pyranose rings
1181	Asymmetric valence vibrations of the C-O-C glycosidic bond
1034	Skeletal vibration of the –CH_2_– group in the ring Asymmetric P–O stretching vibrations in phosphate anions; Ce–O stretching vibrations; P–O–Alk stretching vibrations
826	Deformational vibrations of Ce-O metal-oxygen bonds

## Data Availability

Data available on request due to restrictions. The data presented in this study are available on request from the corresponding author.
